# Mindful Coping Power: Comparative Effects on Children’s Reactive Aggression and Self-Regulation

**DOI:** 10.3390/brainsci11091119

**Published:** 2021-08-25

**Authors:** Caroline L. Boxmeyer, Shari Miller, Devon E. Romero, Nicole P. Powell, Shannon Jones, Lixin Qu, Stephen Tueller, John E. Lochman

**Affiliations:** 1 Department of Psychiatry and Behavioral Medicine, The University of Alabama, Tuscaloosa, AL 35487, USA; 2 Center for Youth Development and Intervention, The University of Alabama, Tuscaloosa, AL 35487, USA; npowell@ua.edu (N.P.P.); jones178@ua.edu (S.J.); lxqu@ua.edu (L.Q.); jlochman@ua.edu (J.E.L.); 3 3605 Moonlight Drive, Chapel Hill, NC 27516, USA; sharipeace@gmail.com; 4 Department of Counseling, University of Texas at San Antonio, San Antonio, TX 78249, USA; devon.romero@utsa.edu; 5 Division for Applied Justice Research, RTI International, Research Triangle Park, Durham, NC 27709, USA; stueller@rti.org; 6 Department of Psychology, The University of Alabama, Tuscaloosa, AL 35487, USA

**Keywords:** mindfulness, reactive aggression, disruptive behavior, Coping Power, self-regulation, prevention, Mindful Coping Power

## Abstract

Coping Power (CP) is an evidence-based preventive intervention for youth with disruptive behavior problems. This study examined whether Mindful Coping Power (MCP), a novel adaptation which integrates mindfulness into CP, enhances program effects on children’s reactive aggression and self-regulation. A pilot randomized design was utilized to estimate the effect sizes for MCP versus CP in a sample of 102 child participants (fifth grade students, predominantly low-middle income, 87% Black). MCP produced significantly greater improvement in children’s self-reported dysregulation (emotional, behavioral, cognitive) than CP, including children’s perceived anger modulation. Small to moderate effects favoring MCP were also observed for improvements in child-reported inhibitory control and breath awareness and parent-reported child attentional capacity and social skills. MCP did not yield a differential effect on teacher-rated reactive aggression. CP produced a stronger effect than MCP on parent-reported externalizing behavior problems. Although MCP did not enhance program effects on children’s reactive aggression as expected, it did have enhancing effects on children’s internal, embodied experiences (self-regulation, anger modulation, breath awareness). Future studies are needed to compare MCP and CP in a large scale, controlled efficacy trial and to examine whether MCP-produced improvements in children’s internal experiences lead to improvements in their observable behavior over time.

## 1. Introduction

Coping Power (CP) is an evidence-based preventive intervention for preadolescent children with disruptive behavior problems [1]. Thirteen randomized controlled trials have shown that CP has beneficial effects for children exhibiting elevated levels of aggressive behavior, producing lower rates of children’s substance use, aggression, and delinquency in later adolescence compared to children in control groups, and in improving children’s social competence and academic functioning (for review, see [1]). Based on a contextual social-cognitive model of risk for aggression and substance use, CP targets mediating child (social cognition, anger coping) and family (parenting) processes [2]. CP’s preventive effects on delinquent behavior and substance use are evident four years after intervention [3]. 

### 1.1. Reactive Aggression

Despite its strong evidence base, CP’s effects have been more mixed on reactive aggression than on proactive aggression. Coping Power had effects on reactive aggression at a three-year follow-up [4], but only had effects on reactive aggression at immediate post-intervention in one of two studies [5,6]. In contrast, Coping Power had significant effects on proactive aggression at both follow-up and immediate post-intervention in these three studies. Reactive aggression is one of two key pathways linking aggression and substance use [7]. Proactive aggression is instrumental, cold-blooded, and unprovoked, whereas reactive aggression is emotionally-driven, impulsive, and hot-blooded [8]. Although children can manifest both forms, factor analytic work consistently finds proactive and reactive aggression to be independent dimensions [9], with unique genetic [10], physiological [11], and social-cognitive processes [12,13]. Proactive and reactive aggression are both important predictors of children’s later substance use and delinquency [14]. The current study was undertaken to maximize CP’s effects on reactive aggression.

Key characteristics that link reactive aggression and substance use include impulsivity and negative emotionality [15,16]. Youths with high levels of reactive aggression may cope with negative emotionality by self-medicating, consistent with research linking temperamental anger to alcohol use initiation [17]. Reactive aggression is also impulsive in nature, and impulsivity has been associated with substance use [15,16]. The current study sought to enhance the effects of CP by targeting the active mechanisms of reactive aggression. 

### 1.2. Active Mechanisms of Reactive Aggression

**Anger arousal and emotional dysregulation.** A central mechanism in reactive aggression is emotional dysregulation [18]. Children with reactive aggression experience high levels of anger [19,20], intense emotional arousal [13,21], and negative emotionality [19,22]. Children high on reactive aggression (but not proactive aggression) evidence greater electrodermal reactivity in response to an experimental anger induction task [11]. 

**Impulsivity and behavioral dysregulation**. Children high on reactive aggression exhibit deficits in behavioral regulation, particularly poor impulse control (e.g., [19]). When they perceive the slightest threat, they lack behavioral inhibition and respond with angry outbursts and aggression [13,23]. Impulsivity is a symptom of attention-deficit hyperactivity disorder (ADHD) and reactive aggression is more strongly associated with ADHD than proactive aggression [24]. 

**Rumination, perceived threat, and cognitive dysregulation**. In ambiguous situations, reactively aggressive children perceive that others have hostile intentions, which leads to angry responses to perceived provocations or threats [9,25]. Anger rumination has been found to be positively associated with reactive aggression in college students [26]. Reactively aggressive children may ruminate about perceived threats and anger-arousing events, which compromises their ability to override tendencies toward aggressive behavior. In an overview of research with adults, this ruminative cognitive style was found to exacerbate anger arousal and create a state of readiness for reactive aggression [27]. Reactive aggression is also linked with deficits in executive function [28]. 

**Attentional capacity**. Children high on reactive aggression often exhibit attention difficulties, as reflected by the higher rates of ADHD in children exhibiting reactive aggression [24]. They also have difficulty accurately encoding social cues and recall fewer details of a social situation [29]. Consequently, reactively aggressive children may miss critical information that informs their responses to others. In addition, their attention is selective and biased, and focuses on negative interactions such as rejection, ridicule, and failure [30]. 

### 1.3. Rationale for Mindful Coping Power

Improving these active mechanisms is expected to reduce children’s reactive aggression and improve their prosocial behavior. In turn, these improvements are expected to disrupt the pathway from reactive aggression to peer rejection, peer delinquency, and substance use. The Mindful Coping Power program (MCP) was developed to maximize program effects on children’s reactive aggression and its active mechanisms, with the overarching aim of altering this developmental cascade. Mindfulness is the practice of bringing non-judgmental awareness to the present moment [31]. Mindfulness training was selected to enhance the existing CP program due to the demonstrated benefits of mindfulness on the active mechanisms of reactive aggression, as described below. 

**Effects of mindfulness on anger arousal and emotion regulation**. Mindfulness is associated with improved emotion regulation and decreased aggressive anger expression in adults [32], college students [33], and children [34]. Brain imaging research with adults and college students indicates that mindfulness training yields improvements in brain regions associated with emotion regulation [35,36]. In studies with youths, mindfulness training has been found to decrease emotional arousal and increase self-efficacy in emotional regulation [34,37].

**Effects of mindfulness on impulse control and behavioral regulation.** Mindfulness training has been found to improve both impulsivity and aggressive behavior in youths with classroom behavior difficulties [38]. A mindfulness-based school program led to improvements in children’s prosocial behavior, aggression, and peer acceptance [39]. Children with ADHD [40,41], children with co-existing ADHD and oppositional defiant disorder [42], and adolescents with disruptive behavior disorders [43] exhibit behavioral improvements following mindfulness training, including reduced hyperactivity and impulsivity. 

**Effects of mindfulness on rumination and cognitive regulation.** Mindfulness training has demonstrated benefits for several aspects of cognitive regulation. It decreases rumination in adolescents [43,44]. In an electroencephalogram study, mindfulness was inversely associated with rumination [45]. The non-judgment component of mindfulness training appears to be relevant for the negative relation between mindfulness and rumination in research with adult participants [46]. Mindfulness training also improves cognitive flexibility with adults [35,47], and with children when implemented in school settings [39]. 

**Effects of mindfulness on attention regulation.** Prior adult studies have shown benefits of mindfulness training on attention (e.g., [48]), including intensive [49] and shorter-term [33] meditation training. Mindfulness improves electrophysiological markers of attentional control [50] and functional connectivity in brain regions important to attention [51] for adults. Important to the current study, Schonert-Reichl and colleagues [39] found that a mindfulness-based school program had positive effects on several behavioral measures of children’s attention and executive function. Further, in two studies of children with ADHD, mindfulness training improved children’s attention [41,42].

**School-based mindfulness intervention.** Although schools can represent an optimal setting for providing mindfulness intervention to a broad range of children, concerns have been raised that the implementation of mindfulness-based interventions is proceeding faster than the current evidence base for school-based implementation documents [52,53,54]. Reviews of the rapidly growing research literature on school-based mindfulness interventions indicate promising effects on attention control, coping with stressors, and, in some cases, on anxiety, but there have been noted concerns about study quality and implementation acceptability (e.g., [55,56]). A systematic review of school-based mindfulness studies concluded that there was insufficient attention to intervention integrity and to feasibility of mindfulness of interventions in school settings [53]. In sum, there is a research base indicating that mindfulness training can lead to improvements in the active mechanisms for reactive aggression in children. The present study was the first to test a school-based mindfulness enhancement of an existing, evidence-based preventive intervention with children with high levels of reactive aggression, with special attention to intervention integrity and to the feasibility and acceptability of the intervention according to intervention providers, children, and parents.

### 1.4. Current Study 

The current study examined whether optimizing CP by infusing it with mindfulness enhances program effects over and above standard CP on children’s reactive aggression and its active mechanisms. A randomized comparative effectiveness trial design [57] was employed to test a previously established “best practice” (CP) against a novel intervention (MCP). The primary study aim was to estimate effect sizes comparing MCP and CP, in preparation for a large-scale efficacy trial. The study had the following a priori hypotheses: (1) MCP will yield greater decreases in children’s reactive aggression than CP; (2) MCP will yield stronger effects than CP on the active mechanisms of reactive aggression, including: decreased child anger arousal and emotional reactivity, increased impulse control and behavioral regulation, decreased anger rumination and cognitive dysregulation, and increased child attentional capacity; (3) MCP will yield greater improvements in children’s social skills than CP, including increased prosocial behavior and decreased externalizing behavior problems. 

## 2. Materials and Methods

### 2.1. Participants

**Participating schools**. Five elementary schools from a public-school system in urban and suburban areas in Alabama were recruited to participate in this study. All schools agreed to participate. The five participating schools varied on sociodemographic measures, including percent of children from economically disadvantaged households (which ranged from 76% to 32%) and child race (Black or African American was most prevalent and ranged from 92% to 32%). Random assignment to condition occurred within school to control for sociodemographic variation.

**Child and parent participants.** This study included a sample of 102 children with elevated levels of reactive aggression, and their parents and teachers. This sample size was selected because it was the maximal number of participants who could be assessed and treated within the project’s grant budget as a pilot and feasibility trial. Power to detect statistically significant differences between the two active interventions was limited. A priori power estimates ranged from 0.21 to 0.62 for small (0.2) to moderate (0.4) effects. This sample size was adequate for the primary aim of estimating the comparative effects of MCP and CP in preparation for a large-scale prevention trial. 

Child participants were identified at the end of fourth grade and participated in the intervention during fifth grade. CP was designed to provide skills-training prior to the middle school transition, when risk for substance use initiation increases (e.g., [58]). To identify children with elevated levels of reactive aggression, fourth grade teachers completed the 3-item reactive aggression scale from the Teacher Report of Reactive and Proactive Aggression [8] on all students in their classroom. Detailed information about this measure is provided below. Screening occurred at the end of fourth grade when teachers were very familiar with children’s behavior. This also allowed families to be recruited and assessed near the start of fifth grade. 

Ratings were compiled across the participating schools to identify an empirical cut-off score reflecting the top quartile of fourth grade students on reactive aggression. A cut-off score of 8 was used. This is consistent with prior studies of CP, in which children with teacher-rated reactive aggression above 8.5 had parent-rated externalizing problems in the at-risk or clinical range on the Behavior Assessment Scale for Children [59]. Thus, teacher screener scores at or above this level are indicative of a child’s risk status from both teacher and parent perspectives [60,61]. Parent participants were the primary caregiver(s) of each child enrolled in the study. 

A total of 638 fourth grade students were screened for study participation. Of those, 428 scored below the empirical cut-off for teacher-rated reactive aggression. One child with an eligible screener score was excluded due to a language barrier that could not be addressed with local resources. The remaining families were contacted in random order until the total number of intervention slots at each school had been filled. Six families declined study participation (the most common reasons were that the child already received services elsewhere, or lack of perceived need). These children did not significantly differ on baseline characteristics from those enrolled. One hundred and eight children were initially enrolled in the study. Five of these children moved to different schools prior to starting fifth grade. The remaining 103 participants were randomly assigned to MCP or CP in yoked pairs, as described below. One child withdrew from the study after participating in one session (due to perceived lack of need and social concerns), which resulted in a total sample of 102 child participants, as well as their parents and teachers. Participants were recruited in two annual cohorts (*n* = 44 in Cohort 1 and *n* = 58 in Cohort 2). 

Table 1 summarizes the demographic characteristics of the study sample. Sixty-one percent of the recruited children were male (*n* = 62) and 39% (*n* = 40) were female. Child age ranged from 9 to 11 (*M* = 9.97, *SD* = 0.48). The parent-reported racial composition of the sample was 87.3% Black or African American (*n* = 89), 5.9% White or Caucasian (*n* = 6), 3.9% more than one race (*n* = 4), and 2.9% Unknown or not reported (*n* = 3). At one of the five schools, the percent of Black or African American children enrolled in the study (70%) was significantly higher than the percent in fifth grade at that school (32%). For family income, 33.4% of parents reported an annual household income of less than USD 15,000, 29.4% reported USD 15,000 to USD 29,999, 21.5% reported USD 30,000 to USD 49,999, 13.8% reported annual family income of more than USD 50,000, and 1.9% did not provide information about annual family income.

**Teachers**. Children’s fourth grade teachers provided initial child behavioral screening data. Children’s fifth grade teachers completed pre- and post-intervention assessments regarding children’s academic and behavioral functioning. The pre-intervention assessment occurred at least four to six weeks after the beginning of fifth grade, to allow time for teachers to get to know the children well. The post-intervention assessment occurred at the end of fifth grade, after the intervention was complete. Teachers were blind to intervention condition (CP or MCP). They only knew that specific children were participating in one of two Coping Power groups being offered at their school (e.g., the Wednesday group or the Friday group). The only other overt difference was that yoga mats were used in MCP but not CP. The yoga mats were stored in a separate meeting room throughout the year, then given to children to keep after all intervention and assessments had been completed. It is possible that children in MCP discussed some of the unique program elements (e.g., yoga stretches, mindfulness practices) with their teachers, but this was not directly evaluated.

**Coping Power leaders**. There were five primary group leaders. Each implemented both versions of the intervention (MCP and CP). These leaders were doctoral (*n* = 2) or master’s (*n* = 3) level clinicians with considerable experience implementing Coping Power (three of the five were licensed clinicians, each with more than twelve years of experience running CP groups; two were advanced doctoral students with prior experience leading or co-leading CP groups). Four of the leaders were female and one male. Four of the leaders were Caucasian and one identified as more than one race. All of the primary leaders completed Mindfulness-Based Stress Reduction training prior to the start of the study. They also committed to maintaining a regular mindfulness practice of at least ten minutes a day throughout the study and participated in weekly group supervision meetings that included group mindfulness practice and discussion. Master’s and advanced undergraduate students served as group co-leaders, to provide additional group oversight and behavior support. These co-leaders participated in CP and mindfulness training workshops prior to program implementation and committed to maintaining a regular personal mindfulness practice.

### 2.2. Procedure

The University of Alabama Institutional Review Board approved all study procedures and conducted continuing review throughout study administration.

**Random assignment to condition**. Children with scores of 8 or higher on the teacher-rated reactive aggression screener items were considered eligible for participation. The participating schools varied in the number of children who fell within the eligible range. A random calling order was created for eligible children from each school and families were contacted according to their placement on this list until twelve children were enrolled at each school. Once recruited into the study, children were randomly assigned to one of the two active conditions: CP (*n* = 50) or MCP (*n* = 52). Random assignment occurred within each school. Yoked pairs of students with similar reactive aggression scores and demographic characteristics (i.e., gender and race) were randomly assigned to either the CP or MCP group at that school. The CP and MCP groups were equivalent on teacher-reported reactive aggression at the end of fourth grade (as shown in Table 1). 

Pre-intervention data (Time 1) were gathered from parents and children near the start of fifth grade and from teachers four to six weeks into the school year. This allowed time to enroll participants while fifth grade teachers were becoming familiar with the children’s behavior (beyond any honeymoon period). The intervention began after all Time 1 assessments were completed. Post-intervention data (Time 2) were collected from teachers in late spring of fifth grade and from parents and children in late spring-early summer after fifth grade. Research staff members who administered the parent and child assessments were blind to condition. Teachers received USD 10 for each child assessed. Parents received USD 50 and child participants received USD 10 at each assessment time point. 

**Intervention**. Two active preventive interventions were compared in this study. CP and MCP both included the same number of sessions (25 child group sessions, 10 parent group sessions) and utilized curriculum manuals with specific objectives for each session. Child group sessions were conducted in a private meeting space during the regular school day and lasted approximately 45 min each. Parent group sessions were held in the morning or evening (both options were offered in each condition) in a location central to the participating schools. Parent group sessions lasted 1–1½ hours each. Sessions were spaced to provide the content in one school year (about 7–8 months). Child groups met weekly and parent groups met 1–2 times per month.

**Coping Power**. CP is an evidence-based preventive intervention for youths with or at risk for disruptive behavior disorders [1]. CP draws upon a cognitive-behavioral framework to teach children social and emotional coping skills and to teach parents positive parenting and self-care skills. Topics covered in the child program include: personal goal setting, identification of feelings, coping with anger, perspective-taking, problem-solving, affiliating with prosocial peers, and resisting peer pressure. Topics covered in the parent program include: supporting children’s academic learning, strengthening the parent–child relationship and family cohesion, managing the stress of parenting, setting household rules and expectations, praise, ignoring, effective discipline techniques, family problem-solving, and planning for the future. 

**Mindful Coping Power**. MCP is a novel adaptation of CP in which mindfulness practices were integrated with the existing cognitive-behavioral elements. All of the core content from the CP child and parent components was retained in MCP. Mindfulness practices were integrated into CP in three ways: (a) mindfulness-only sessions (several sessions were added to the MCP child and parent programs to introduce mindfulness theory and practice); (b) mindfulness in every session (each MCP child and parent session began and ended with a series of mindfulness practices, including the ringing of a chime, a breath awareness practice, yoga poses, and a compassion practice); (c) integration of mindfulness into existing Coping Power activities (e.g., an existing component on identifying early physiological cues of anger was enhanced through regular body awareness practices; compassion practices informed activities designed to help children and parents see situations from others’ perspectives; thought awareness practices helped children and parents allow angry thoughts to ‘pass on by’ rather than clinging to them as facts). For further detail about the integration of mindfulness into CP, including the comprehensive theoretical model for MCP and sample sessions highlighting the differences between MCP and CP, see Miller and colleagues [62]. 

Every child and parent intervention session (CP and MCP) was video recorded. Group leaders received monthly individualized feedback as well as weekly group supervision from the principal investigators. To ensure high fidelity to the new program elements, leaders received feedback on every mindfulness-only session held. Leaders were trained to ensure that they did not incorporate mindfulness-specific language or practices into CP sessions. CP did not have any intervention content that was not also in MCP. Effort was made to keep the length of sessions consistent across the two conditions. Due to the added mindfulness practices in MCP, leaders were able to spend more time on some program elements during CP sessions than MCP sessions (e.g., reviewing progress toward weekly personal goals and setting new goals; personal sharing related to intervention topics; opportunities to practice new skills in session; summarizing key points at the end of sessions). There were a total of 18 unique child groups (9 MCP, 9 CP) and 8 unique parent groups (4 MCP, 4 CP). Four schools participated in Cohort 1 and a fifth school was added for Cohort 2. The same leader ran both the MCP and CP groups at each school each year. 

### 2.3. Measures

**Teacher Report of Reactive and Proactive Aggression** (TRRPA) [8]. To identify students with moderate to high levels of reactive aggression, fourth grade teachers completed the 6-item TRRPA for all of the children in their class. This instrument consisted of three items assessing reactive aggression (“overreacts angrily to accidents,” “strikes back when teased,” and “blames others in fights”) and three items assessing proactive aggression (“gets others to gang up on a peer,” “uses physical force to dominate,” and “threatens and bullies others”). Each item was rated on a 5-point scale (1 = *Never* to 5 = *Almost Always*). Children’s scores on the 3-item teacher-reported reactive aggression scale were used to determine study eligibility (scores can range from 3 to 15, with higher scores reflecting greater reactive aggression). Teachers also completed this measure pre- and post-intervention to assess for change. In the current sample, Cronbach’s alpha for teacher-rated reactive aggression was 0.91. 

**Abbreviated Dysregulation Inventory** (ADI) [63]. The 31-item ADI includes three subscales: affective dysregulation (arousability, weak emotional control, and irritability); behavioral dysregulation (impulsivity, inattention, and hyperactivity); cognitive dysregulation (poor problem-solving and planning, inability to learn from experience, and cognitive inflexibility). Students rated how true each statement was in the past month on a Likert-type scale from 1 (*never true*) to 4 (*always true*). Total dysregulation was calculated by averaging the scores for all three subscales, with higher scores reflecting greater overall dysregulation. Five of the affective dysregulation items comprise the Anger Scale (e.g., “I have trouble controlling my temper,” “when I am angry I lose control over my actions,” “I get so frustrated that I often feel like a bomb ready to explode), with higher scores indicating greater difficulty regulating anger. This well-established instrument has been used with children and adults and is used to assess risk for substance use disorders. In the current sample, Cronbach’s alpha was 0.81 for total dysregulation and 0.74 for the anger scale. 

**Early Adolescent Temperament Questionnaire** (EATQ) [64]. The EATQ is a self-report measure that assesses various aspects of adolescent temperament [64]. The inhibitory control subscale of the EATQ was administered as a child self-report measure of behavioral self-regulation. This subscale consists of five items (e.g., “when someone tells me to stop doing something, it is easy for me to stop”). Higher mean scores reflect a greater capacity to plan and suppress inappropriate responses. Cronbach’s alpha for the EATQ inhibitory control subscale was 0.63 in the current sample. The attention subscale of the EATQ-Revised Parent Report (EATQ-R) [65] was administered as a parent-report measure of children’s attentional capacity. This subscale consists of six items (e.g., “my child finds it really easy to concentrate on a problem,” “my child is good at keeping track of several different things that are happening around him/her”). Higher mean scores reflect better child attentional capacity. Cronbach’s alpha for the EATQ-R attention subscale was 0.73 in the current sample. 

**Behavior Assessment System for Children** (BASC) [59]. Child externalizing behavior problems and social skills were assessed using the Parent Rating Scale (PRS) and Teacher Rating Scale (TRS) of the BASC. Items of the PRS and TRS were rated on a 4-point scale (e.g., “Mean to others,” “Sudden changes in mood or feelings”; 0 = *Never* to 3 = *Almost Always*). The child version (appropriate for ages 6–11) was used for both the TRS and PRS. Teachers and parents completed the BASC at pre- and post-intervention time points. 

Program effects on children’s aggressive and disruptive behavior were measured using the externalizing problems composite, which consists of subscales measuring aggression, conduct problems, and hyperactivity. Higher composite scores reflect worse child externalizing problems. Cronbach’s alpha for the externalizing problems scale was 0.89 for the PRS and 0.96 for the TRS in the current sample. The social skills subscale was also included as a measure of program effects on children’s prosocial behavior. Sample items include “offers to help others,” “shows interest in others’ ideas,” and “tries to bring out the best in others.” Higher scores reflect better child social skills. Cronbach’s alpha for the social skills subscale was 0.89 for the PRS and 0.92 for the TRS. Parent and teacher reports of children’s externalizing behavior problems (*r* = 0.34) and social skills (*r* = 0.20) were significantly correlated but represent unique perspectives, thus were considered separately in analyses. 

**Scale of Body Connection** (SBC) [66]. Breath awareness was examined as a measure of mindfulness. Four items assessing children’s breath awareness were adapted based on the SBC [61], which includes a range of items measuring body awareness and bodily dissociation. Items were rated on a 1 (*not at all*) to 5 (*all of the time*) Likert scale. Sample items included “I can feel my breath travel through my body” and “I notice how my breath changes when I am tense or nervous.” Higher mean scores reflect greater child awareness of their breath. Cronbach’s alpha for the breath awareness scale in the current sample was 0.65. 

**Program Implementation**. Child and parent group leaders documented program implementation in several ways. For each CP and MCP session, they documented participant attendance, level of engagement, and completion of in- and out-of-session activities. They also documented the duration of each session and completion of the planned objectives. Supervisors also rated leaders’ implementation fidelity and quality using program-specific measures (assessing completion of planned program objectives and constructs such as: ability to engage participants in the intervention, effective instructional style, and consistency with guiding theoretical principles). 

**Program Feasibility, Acceptability, and Impact**. Participants provided feedback on MCP and CP acceptability and impact after every 5 child group sessions and every 2 parent group sessions, and provided overall feedback at post-intervention. Group leaders provided feedback on the feasibility and impact of each MCP and CP session and overall feedback at post-intervention.

### 2.4. Data Analyses

Latent Change Score (LCS) analyses were conducted to compare CP and MCP intervention effects on the primary child outcome measures. LCS is a structural equation model (SEM) that can be used to fit the paired *t*-test model in a way that is more flexible than the usual paired *t*-test [67]. For the current data, this allowed multiple group *t*-tests to be fit and to examine whether there were pre–post outcome changes within the standard and mindful Coping Power groups, and whether the amount of change differed between the CP and MCP groups (this can be seen as an SEM implementation of the difference-in-differences estimator). 

The multiple group latent change scores were fit using the lavaan [68] R package [69]. Models were fit such that pre-test values predicted the latent change score, which allows for the likely possibility of imperfect test-retest reliability [67]. The LCS approach also has better missing data handling than the paired *t*-test since it assumes data are missing at random (MAR) conditional on other variables in the model instead of the missing completely at random (MCAR) assumption made by the *t*-test, which excludes all participants with any missing data. For a review of these missing data concepts, see Enders [70]. Full information maximum likelihood estimation was used to address missing data so that all participants were retained in the analyses. All participants with an observation for at least one time point are retained. At pre-intervention, 1 of 52 MCP participants and 1 of 50 CP participants were missing data on variable(s) of interest. At post-intervention, 3 MCP and 8 CP participants were missing data on variable(s) of interest. Before conducting LCS analyses, *t*-tests were run to compare MCP and CP for baseline equivalence on each of the primary child outcome measures. The distribution of scores on each measure was also examined for skewness and kurtosis.

The focus of the analyses was on estimating effect sizes in MCP and CP to inform power analyses for a future, large-scale efficacy trial. It was expected that preliminary support for MCP would be evidenced by at least small to moderate effect sizes relative to CP on the primary outcomes (*d* = 0.2 or larger), which would warrant further evaluation of MCP in a large-scale efficacy trial.

## 3. Results

LCS analyses compared the effects of the new MCP preventive intervention to the effects of the existing, evidence-based CP program on reactive aggression and its active mechanisms. Table 2 summarizes the findings observed. Results are presented by informant group, with a focus on the effect size estimates observed.

### 3.1. Child Self-Report Outcomes

**Figure 1:**
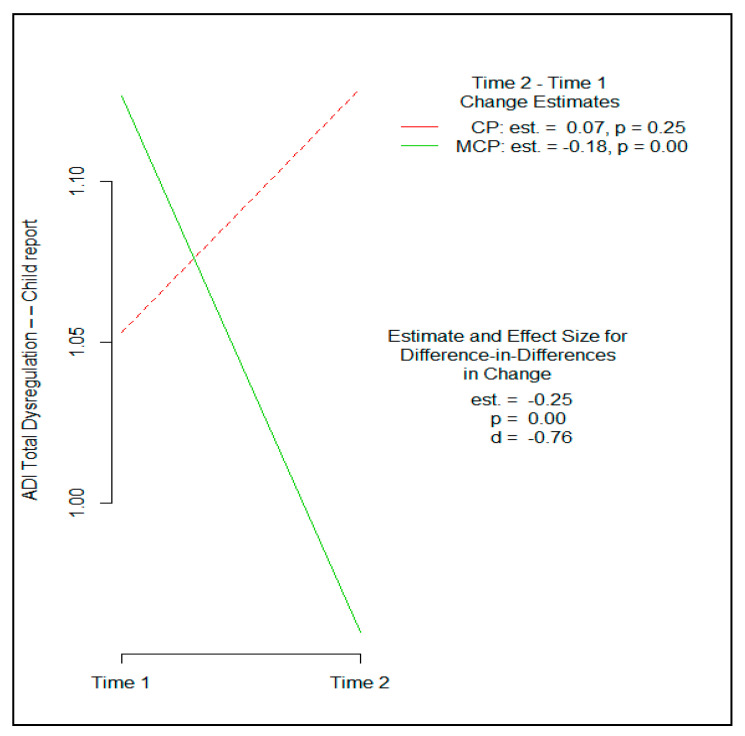
Comparative effects of Coping Power and Mindful Coping Power on children’s total dysregulation (affective, behavioral, cognitive) on the Abbreviated Dysregulation Inventory.

Children in MCP exhibited a significantly greater reduction in total dysregulation on the ADI compared to children in CP, yielding a moderate–large effect size (Cohen’s *d* = −0.76, 95% CI [−1.40, −0.10], *p* = 0.001). Figure 1 depicts this finding. 

Moderate effect sizes favoring MCP were observed on the affective dysregulation (Cohen’s *d* = −0.42, 95% CI [−0.53, −0.003], *p* = 0.048) and behavioral dysregulation (Cohen’s *d* = −0.41, 95% CI [−0.52, 0.001], *p* = 0.051) subscales of the ADI. A smaller effect size was observed on the cognitive dysregulation subscale (Cohen’s *d* = −0.33, 95% CI [−0.47, 0.04], *p* = 0.102), also favoring MCP. Only the effect on affective dysregulation was statistically significant in this feasibility sample. Children in MCP exhibited a significantly greater reduction on the ADI Anger Scale than children in CP, yielding a moderate effect size (Cohen’s *d* = −0.45, 95% CI [−0.70, −0.03], *p* = 0.033). 

**Figure 2:**
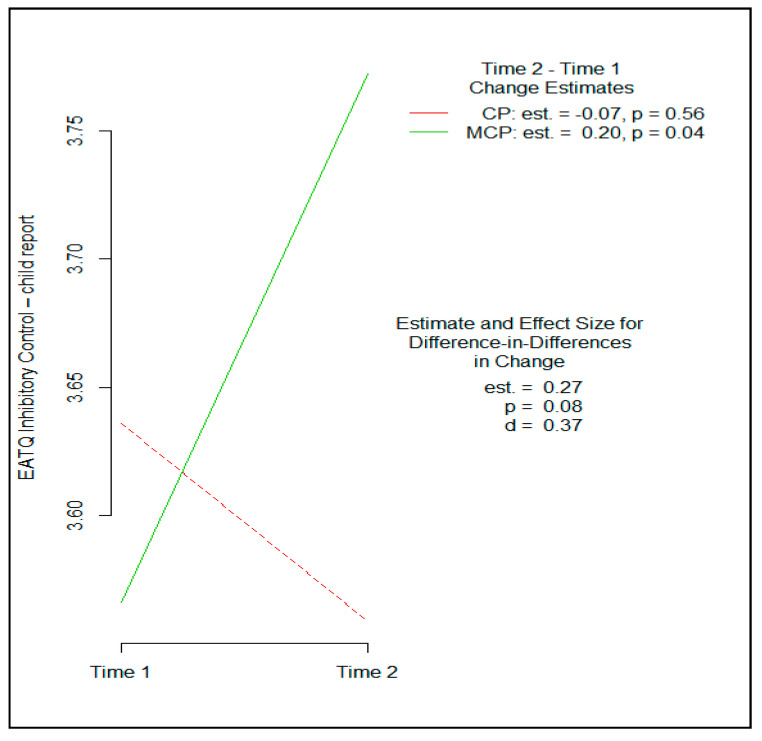
Comparative effects of Coping Power and Mindful Coping Power on children’s inhibitory control on the Early Adolescent Temperament Questionnaire.

A small to moderate effect size favoring MCP was observed for child-reported inhibitory control on the EATQ (Cohen’s *d* = 0.37, 95% CI [−0.03, 0.58], *p* = 0.081). This finding is depicted in Figure 2. Breath awareness, a measure of child mindfulness, also yielded a small to moderate effect size favoring MCP (Cohen’s *d* = 0.31, 95% CI [−0.07, 0.99], *p* = 0.090). These effects were not statistically significant in this feasibility sample. 

### 3.2. Parent-Report Outcomes

Small to moderate effect sizes favoring MCP were observed for parent-reported improvements in child attention on the EATQ (Cohen’s *d* = 0.32, 95% CI [−0.07, 0.58], *p* = 0.121) and children’s social skills on the BASC (Cohen’s *d* = 0.30, 95% CI [−0.96, 5.79], *p* = 0.161). In contrast, there was a small to moderate effect favoring CP for reduction in parent-reported BASC externalizing problems (Cohen’s *d* = 0.36, 95% CI [−0.42, 8.25], *p* = 0.077). No parent-reported outcome effects were statistically significant in this feasibility sample.

### 3.3. Teacher-Report Outcomes

On teacher-rated child reactive aggression, a key outcome of interest, the difference between MCP and CP was small and non-significant (Cohen’s *d* = 0.13, 95% CI [−1.17, 1.51], *p* = 0.802). Negligible differences between MCP and CP were also observed on the two other teacher-reported outcomes, child externalizing problems on the BASC (Cohen’s *d* = −0.04, 95% CI [−5.61, 1.62], *p* = 0.279) and child social skills on the BASC (Cohen’s *d* = −0.02, 95% CI [−3.55, 2.04], *p* = 0.598). 

### 3.4. Implementation Fidelity and Quality

Supervisors who observed video-recorded sessions provided overall ratings of leaders’ implementation fidelity and quality, with scores ranging from 1 (low) to 10 (high). Mean supervisor-rated effective completion of session objectives was 8.33 (*SD* = 0.89) for CP and 8.44 (*SD* = 0.99) for MCP. Mean supervisor-rated effective engagement of participants was 8.67 (*SD* = 0.89) for CP and 9.00 (*SD* = 1.78) for MCP. Supervisors also rated MCP leaders’ ability to articulate mindfulness theory clearly and accurately, yielding a mean score of 8.78 (*SD* = 1.23) and ability to implement mindfulness practices effectively, yielding a mean score of 8.44 (*SD* = 1.75).

### 3.5. School and Leader Effects

Exploratory analyses assessed for school-level effects. No significant effects were observed at the school level. Leader effects were on the same level, since random assignment to condition occurred within schools (i.e., the same leader implemented both the CP and MCP groups at each school). No significant effects at this level were observed. 

### 3.6. Feasibility and Participant Satisfaction

Leaders provided feedback on the comparative feasibility of MCP and CP at post-intervention. On a scale ranging from 0 (not at all) to 10 (very much), the mean score for how feasible the MCP child program was to implement was 7.29 (*SD* = 0.76) and the mean score for how well the leader was able to cover the core skills from standard CP during MCP child sessions was 7.71 (*SD* = 1.28). When asked to rate which program was easier to implement, the mean score was 4.57 (*SD* = 2.30), on a scale ranging from 0 (CP) to 5 (equal) to 10 (MCP). The following quote summarizes the most prevalent themes in the leader feedback regarding the comparative feasibility of MCP versus CP: “CP had fewer topics to cover in each session, so it was easier to implement in that way. However, as the program went on, the MCP group became calmer and easier to manage. So, even though there was more to cover in any given session, it became somewhat easier to implement overall.”

On post-intervention feedback surveys, children rated how helpful the MCP or CP program was to their life. Children’s mean rating for MCP was 3.5 (*SD* = 0.7) compared to 3.3 (*SD* = 1.2) for CP on a 4-point Likert scale ranging from 1 (Not at all) to 4 (Very much). Children rated how well they liked being in the program on the same scale, which yielded a mean score of 3.6 (*SD* = 0.5) for MCP compared to 3.3 (*SD* = 1.1) for CP. Some of the children’s comments about what they liked best about MCP were: “everything,” “it helps you work on your friendships and stuff,” “MCP helps you with your heart,” “we learn things that help us take care of our problems,” “the mindful stuff,” “being normal and calm,” “it helps me handle my feelings,” “PTP and Take 2,” “it teaches you to respond not react,” and “I liked doing Feel and Spread the Good Vibes.” Some of the children’s suggested changes to improve MCP were “nothing,” “that we have more time,” “do it every day,” “to add more people to the group,” “stay longer,” “do more fun stuff,” “the timing [of group meetings at school],” and “get more points and prizes.”

Without the same time constraints for the parent group meetings, leaders found the MCP parent group to be equally feasible to implement as CP, with a mean score of 5.00 (SD = 0) on a scale ranging from 0 (CP) to 5 (equal) to 10 (MCP). Leaders reported that it was very feasible to implement the MCP parent group, with a mean rating of 9.5 out of 10 (SD = 0.58). They also reported that they were readily able to cover the standard CP topics in the parent MCP group, with a mean of 10.0 (SD = 0).

Parents who participated in MCP parent meetings were very receptive to the program. The following quotes represent common feedback from parents about the MCP parent program: “parents and children should participate to learn how to handle situations by thinking before they react and pay attention to how they cope with different situations;” “I really enjoyed each session because every session was growth and knowledge about solving problems effectively, communicating more calmly, and becoming aware of how to deal with certain situations.” Recommended improvements focused on maintaining the supportive community, e.g., “we should have a dinner or picnic,” and “we should stay in touch and keep supporting each other” as well as practical scheduling matters.

## 4. Discussion

This comparative effectiveness study examined whether MCP leads to greater improvements in child reactive aggression and its active mechanisms than CP, with the primary goal of estimating effect sizes in advance of a large-scale efficacy trial. CP is an evidence-based preventive intervention for children with disruptive behavior problems. Thus, the effect-size estimates observed provide valuable information regarding potential enhancing effects of MCP for children with disruptive behavior problems. Findings varied by informant group, as discussed below. 

### 4.1. Child Self-Report Findings 

MCP yielded stronger effects than CP on children’s self-reported total dysregulation on the ADI, as well as on each of the ADI’s subscales (emotional, behavioral and cognitive dysregulation). This is noteworthy for several reasons. First, it provides support for the hypothesis that integrating mindfulness into CP would strengthen program effects on the active mechanisms of reactive aggression, including anger arousal and emotion regulation, impulsivity and behavior regulation, and anger rumination and cognitive regulation. This is also important because past studies have shown that children’s scores on the ADI during preadolescence account for a significant proportion of the variance in substance use at later ages [71]. Given the important role of anger arousal in reactive aggression, scores on the ADI anger scale were examined separately. Despite limited statistical power in this feasibility trial, MCP produced significantly greater improvements in children’s anger modulation on the ADI than CP. Improved anger modulation is also expected to contribute to longer-term beneficial effects across the developmental cascade. 

Other child self-report outcomes provided further support for the study hypotheses. Small to moderate effect sizes favoring MCP were also observed on children’s self-reported inhibitory control and breath awareness. EATQ inhibitory control was included as a separate measure of behavioral self-regulation, which is often deficient in children who exhibit high levels of reactive aggression. Thus, MCP was found to strengthen CP’s effects across two different child-report measures of inhibitory control, the ADI and EATQ. 

Breath awareness was included as a measure of children’s present moment and embodied mindful awareness. In MCP, there was especially good uptake of a specific breath awareness practice called “Press the Pause and Take Two Breaths” (PTP and Take 2). Once this brief breath awareness practice was introduced, children in MCP were readily able to recall and practice it on their own. They shared real life situations in which they noticed a need for a calming, centering activity and opted on their own to practice PTP and Take 2. Breath-centered meditation practices have many health benefits, which Melnychuk and colleagues [72] attribute to the coupling of respiration and attention. Specifically, focusing on and regulating breathing can optimize attention, and likewise, focusing one’s attention leads breathing to become more synchronized.

### 4.2. Parent-Report Findings 

Parent-report outcomes yielded mixed results. Small to moderate effect sizes favoring MCP were observed on two parent-reported outcomes. The first was child attention, as measured on the EATQ. This finding supports the hypothesis that adding mindfulness to CP would enhance program effects on the active mechanisms of reactive aggression, including children’s attentional capacity. As described above, reactive aggression is more strongly associated with attentional difficulties than proactive aggression, including the diagnosis of ADHD [24]. The current findings indicate that incorporating mindfulness into CP has a small to moderate enhancing effect on attentional capacity in children with reactive aggression (based on parent observations). Better present moment awareness and attentional control may help children encode social cues more accurately and relate better with others.

A small to moderate effect size favoring MCP was also observed for children’s social skills, as measured by parent-report on the BASC. Children with reactive aggression often have both attentional and social difficulties, which are interrelated. Children with reactive aggression have difficulty accurately encoding social cues and recall fewer details of a social situation [29]. Consequently, reactively aggressive children may miss critical information that informs their responses to others. In addition, their attention is selective and biased, and focuses on negative interactions such as rejection, ridicule, and failure [30] and perceived threats. The current findings indicate that MCP enhances CP’s effects on children’s social skills (based on parent observation). Future research can explore the extent to which social skill improvements are related to interpersonal skills taught in the program versus improved attention. 

One of the mindfulness enhancements in MCP was the practice of extending compassion to self and others. This practice, Feel and Spread the Good Vibes, was repeated at the end of every MCP session (and was not included in CP). The addition of repeated compassion practices may have contributed to MCP’s enhanced effects on children’s prosocial behavior. Reactive aggression is linked to substance use through a complex mediational chain, in which reactive aggression leads to peer rejection, peer rejection leads to peer delinquency, and peer delinquency leads to substance use [7]. Improving children’s social skills may disrupt this trajectory by reducing peer rejection and delinquency. Thus, it is important to maximize program effects on children’s social skills and peer acceptance. Long-term follow-up is needed to assess the impact of MCP across this developmental cascade. 

An unexpected finding was a small to moderate effect size favoring CP for parent-reported child externalizing problems on the BASC. Although the opposite effect was hypothesized, there are some possible explanations for this finding. In an effort to balance the amount of home practice in MCP and CP, daily home behavior goals were added to CP (the original CP program only includes school behavior goals). Children in CP set a personal home behavior goal every week (e.g., complete my chores with a positive attitude, get along better with my sister, follow directions the first time) and earned points for completing these goals. Many children in CP put considerable effort into accomplishing these home behavior goals. This may have been a more “active ingredient” than anticipated, contributing to more observable behavioral improvement at home in CP at post-intervention, relative to the corresponding home mindfulness practices completed by children in MCP. 

The MCP and CP conditions may have also differed (unintentionally) in the amount of time spent on discussion and practice of cognitive-behavioral skills. MCP was designed to include all of the same cognitive-behavioral topics as CP, plus additional opening and closing mindfulness practices. Child session content was planned for 50–60 min; however, many schools asked for the child groups to be held over lunch, which allowed for sessions lasting closer to 35–45 min (for both CP and MCP). In MCP, it was important for the mindfulness practices at the beginning and end of each session to be led in an unhurried way. This left less time in the middle of some MCP sessions for discussion and practice of new cognitive-behavioral skills. Leaders reported that they were able to cover all of the core intervention content in MCP, but had more limited opportunities to discuss and practice new cognitive-behavioral skills than in CP. It is possible that this diminished MCP effects on observable child behavioral outcomes. This can be addressed in the future by holding sessions that last the full 50–60 min (as planned), or by modifying the content to be offered in a larger number of sessions that meet for 35–45 min each, which may work better in school settings. 

### 4.3. Teacher-Report Findings

Teacher-report data yielded no effect sizes in the small (*d* = 0.20) or larger range that differentiated the outcomes observed in MCP and CP. This included teacher-rated reactive aggression, which was a primary outcome variable of interest (and the measure upon which children were screened for the intervention). This also included teacher-rated child externalizing problems and social skills on the BASC. Thus, the hypothesis that MCP would enhance the effects of CP on reactive aggression and other teacher-reported child behavioral outcomes was not supported. Teachers are valuable informants given their experience interacting with a wide range of children and the frequent opportunities they have to observe children’s behavior and social interactions throughout the school day. 

Of note, children’s teacher-reported BASC externalizing problems worsened from pre- to post-intervention in both MCP and CP. This may relate to the timing of the pre- and post-intervention assessments, which were collected near the beginning and end of the school year, in contrast to the typical format of prior Coping Power studies where the pre-intervention assessment has been collected one spring and post-intervention the following spring. Teachers may have less information about children’s problems in a fall assessment. A prior CP adaptation study with the same fall-to-spring assessment schedule also found that children in both the intervention and control groups had increases in teacher-rated behavior problems from fall-to-spring. However, the children in CP had significantly less increase in conduct problems than control children [73]. This pattern can be better examined in study designs with a no-treatment comparison condition, which the current study did not include. Designs with no-treatment control conditions also help measure whether preventive interventions attenuate increases in behavioral and academic problems across the transition to adolescence in at-risk youths, even if they do not lead to overall net improvements. Although prior research found that CP yielded teacher-rated behavioral improvement for children in comparison to an untreated randomized control group, CP did not influence more trait-like dysregulation scores on the ADI [5]. Thus, MCP has the potential advantage of producing broader generalized changes in children’s ability to self-regulate their behavior and affect relative to CP and no treatment. These are important opportunities for future research. 

### 4.4. Implementation Findings 

This study addresses the need for school-based mindfulness studies with rigorous attention to intervention integrity and to assessing the feasibility of mindfulness interventions in school settings [53]. A rigorous approach was taken in this study to maintain intervention integrity and to assess comparative feasibility from multiple stakeholders’ perspectives. The study provides strong support for the feasibility and uptake of the MCP child and parent programs overall. As described above, modifications can be made to the reduce the amount of content in each MCP child session, to better match the school schedule and improve program feasibility.

### 4.5. Limitations and Future Research

The current study’s comparative effectiveness design was rigorous in that it compared two active interventions in a randomized design. CP is a well-established preventive intervention that has exhibited positive effects in 13 randomized trials, including long-term follow-up. Thus, improving upon CP’s effects in targeted areas is noteworthy. Given the feasibility nature of this study, statistical power was only sufficient to detect effects in the medium to large range. Overall, the current study met its aim of generating effect size estimates to inform a future large-scale efficacy study comparing MCP and CP. 

Participants in the current sample were predominantly Black and low-to-middle income, reflecting the region in which the study was conducted. This is both a strength, in that this study adds to our understanding of the effects of mindfulness-based interventions in racially and economically disadvantaged samples, and a limitation, because future studies are needed to understand the generalizability of this study’s findings in more diverse samples. A specific concern in this study is that there may have been bias in the teacher behavior ratings used to identify at-risk students, particularly at one school in which Black/African American students were overrepresented relative to the overall school population. Screening procedures should account for possible teacher bias, such as by including a second parent-gate screening. 

Future studies would also benefit from collecting long-term follow-up data to examine whether program benefits are sustained beyond post-intervention and impact later outcomes in the developmental cascade, including peer delinquency and substance use. Despite the benefits of MCP on several child-reported outcomes, neither parent- nor teacher-report outcomes yielded the hypothesized enhancing effects of MCP on child aggression or externalizing behavior. One explanation for this pattern of results is that it may take time for the improvements in MCP children’s internal experiences of emotional, behavioral, and cognitive self-regulation to manifest into improvements in aggression and externalizing behavior that are clearly discernable to parents and teachers. In the future, it would be beneficial to conduct longer-term follow-up assessments to assess for this. It would also be valuable to include direct child behavioral assessments to complement child self-report data. 

Since parents were also direct participants in MCP and CP, it will be important to examine program effects on parent-specific outcomes. Innovative study designs could also unpack the relative contributions of various skill-training components included in the comprehensive MCP and CP interventions for children and parents (i.e., attention training, compassion training, goal setting, emotional awareness, thought awareness, problem-solving, assertive communication, mindful parenting, and mindful communication). A precision medicine approach could be applied to identify specific subgroups who would benefit most from specific skill-training components. 

Other important questions in the mindfulness literature are how much practice (frequency, quantity) is needed to yield meaningful benefits, and whether secularized forms of mindfulness practice (such as those that can be taught within public-school settings) have as much benefit as traditions in which these practices are couched within a broader lifestyle and ethic. The overarching principles taught in CP are that everyone experiences difficult emotions and difficult situations, and it is important to be aware of these experiences and to practice ways of coping with them that are compassionate toward oneself and others. While this was consistent across both MCP and CP, the MCP program offered more first-person, in-depth learning opportunities. Future studies can examine whether dosage of practice (both in and out of sessions) and leader embodiment of mindfulness impact short- and long-term program effects. 

## 5. Conclusions

This study provides a valuable contribution to the literature by estimating the comparative effects of MCP, a mindfulness-enhanced adaptation of the Coping Power preventive intervention, to standard CP in a rigorous randomized trial. MCP had the strongest comparative effects on child-reported self-regulation and anger modulation, varied effects on parent-reported outcomes, and minimal effects on teacher-reported outcomes (including child reactive aggression).

## Figures and Tables

**Table 1:** Participant characteristics at time of recruitment.

Demographic variable	Overall Sample (*n* = 102)	MCP Condition (*n* = 52)	CP Condition (*n* = 50)
	*M (SD)*	*M (SD)*	*M (SD)*
Child age	9.97 (0.48)	10 (0.49)	9.94 (0.47)
Child 4th grade reactive aggression	11.18 (2.37)	11.17 (2.39)	11.18 (2.37)
	*n* (%)	*n* (%)	*n* (%)
Child gender			
Male	62 (60.8%)	33 (63.5%)	29 (58.0%)
Female	40 (39.2%)	19 (36.5%)	21 (42.0%)
Child ethnicity			
Hispanic or Latino	3 (2.9%)	1 (1.9%)	2 (4.0%)
Not Hispanic or Latino	92 (90.2%)	47 (90.4%)	45 (90.0%)
Unknown or not reported	7 (6.9%)	4 (7.7%)	3 (6.0%)
Child race			
Black or African American	89 (87.3%)	47 (90.4%)	42 (84.0%)
White or Caucasian	6 (5.9%)	2 (3.8%)	4 (8.0%)
More than one race	4 (3.9%)	1 (1.9%)	3 (6.0%)
Unknown or not reported	3 (2.9%)	2 (3.8%)	1 (2.0%)
Child repeated grade	18 (17.5%)	9 (17.3%)	9 (18.0%)
Caregiver relation to child			
Biological parent	85 (82.5%)	44 (84.6%)	41 (82%)
Adoptive parent	5 (4.9%)	3 (5.8%)	2 (3.8%)
Grandparent	7 (6.8%)	2 (3.8%)	5 (10%)
Other	5 (4.9%)	3 (5.8%)	2 (3.8%)
Annual family income			
Less than USD 15,000	34 (33.4%)	17 (32.7%)	17 (34.0%)
USD 15,000 to <29,999	30 (29.4%)	13 (25.0%)	17 (34.0%)
USD 30,000 to <49,999	22 (21.5%)	14 (26.9%)	8 (16.0%)
More than USD 50,000	14 (13.8%)	7 (13.5%)	7 (14.0%)
Unknown or not reported	2 (1.9%)	1 (1.9%)	1 (2.0%)

**Table 2:** Comparative effects of standard Coping Power (CP) and Mindful Coping Power (MCP) on child outcomes in a pilot randomized trial.

	Means				Latent Change Score (LCS)				Group Differences in LCS	
	Standard Coping Power		Mindful Coping Power		Standard Coping Power		Mindful Coping Power			
	Pre	Post	Pre	Post	Estimate	*p*	Estimate	*p*	Cohen’s *d* (95% CI)	*p*
Child-reported outcomes										
Total Dysregulation (ADI) ^a^	1.05	1.13	1.13	0.96	0.07	0.248	−0.18	0.000	−0.76 (−1.40, −0.10)	0.001
Anger Scale (ADI) ^a^	1.02	1.09	1.07	0.80	0.08	0.540	−0.28	0.010	−0.45 (−0.70, −0.03)	0.033
Inhibitory Control (EATQ) ^b^	3.64	3.56	3.57	3.77	−0.07	0.558	0.20	0.043	0.37 (−0.03, 0.58)	0.081
Breath Awareness (SBC) ^b^	3.33	3.37	3.11	3.64	0.07	0.702	0.53	0.008	0.31 (−0.07, 0.99)	0.090
Parent-reported outcomes										
Attention (EATQ) ^b^	3.28	3.16	3.08	3.23	−0.09	0.326	0.16	0.228	0.32 (−0.07, 0.58)	0.121
Social Skills T-score (BASC) ^b^	53.78	52.05	51.06	51.63	−1.77	0.149	0.65	0.593	0.30 (−0.96, 5.79)	0.161
Externalizing Problems T-score (BASC) ^a^	52.57	49.80	51.98	53.18	−2.81	0.104	1.11	0.422	0.36 (−0.42, 8.25)	0.077
Teacher-reported outcomes										
Reactive Aggression (TRRPA) ^a^	9.40	10.08	9.80	10.56	0.69	0.215	0.87	0.029	0.13 (−1.17, 1.51)	0.802
Social Skills T-score (BASC) ^b^	41.16	44.14	39.87	42.00	2.81	0.013	2.06	0.019	−0.02 (−3.55, 2.04)	0.598
Externalizing Problems T-score (BASC) ^a^	57.78	61.59	58.58	60.74	4.12	0.001	2.13	0.021	−0.04 (−5.61, 1.62)	0.279

^a^ Lower scores reflect positive outcomes. ^b^ Higher scores reflect positive outcomes.

## Data Availability

The data presented in this study are available upon request from the corresponding author. The data will be made publicly available after all planned grant analyses have been published.
